# Antidiabetic activity of *Cassia occidentalis* (Linn) in normal and alloxan-induced diabetic rats

**DOI:** 10.4103/0253-7613.68422

**Published:** 2010-08

**Authors:** Laxmi Verma, Anirudh Khatri, Basant Kaushik, Umesh K. Patil, Rajesh S. Pawar

**Affiliations:** Department of Pharmacology, I.S.F College of Pharmacy, Moga - 142 001, Punjab, India

**Keywords:** Alloxan, antidiabetic activity, *Cassia occidentalis*

## Abstract

**Objective:**

To evaluate the hypoglycemic activity of various extracts, petroleum ether, chloroform and aqueous extract of *Cassia occidentalis* in normal and alloxan-induced diabetic rats.

**Materials and Methods:**

Petroleum ether, chloroform and aqueous extract of whole plant of *Cassia occidentalis* were orally tested at the dose of 200 mg/kg for hypoglycemic effect in normal and alloxan-induced diabetic rats. In addition, changes in body weight, serum cholesterol, triglyceride and total protein levels, assessed in the ethanol extract-treated diabetic rats, were compared with diabetic control and normal animals. Histopathological observations during 21 days treatment were also evaluated.

**Results:**

Aqueous extract of *C. occidentalis* produced a significant reduction in fasting blood glucose levels in the normal and alloxan-induced diabetic rats. Apart from aqueous extract, petroleum ether extract showed activity from day 14 and chloroform extract showed activity from 7 days. Significant differences were observed in serum lipid profiles (cholesterol and triglyceride), serum protein, and changes in body weight by aqueous extract treated-diabetic animals, when compared with the diabetic control and normal animals. Concurrent histopathological studies of the pancreas of these animals showed comparable regeneration by extract which were earlier necrosed by alloxan.

**Conclusion:**

Aqueous extract of *C. occidentalis* exhibited significant antihyperglycemic activity in normal and alloxan-induced diabetic rats. They also showed improvement in parameters like body weight and serum lipid profiles as well as histopathological studies showed regeneration of β-cells of pancreas and so might be of value in diabetes treatment.

## Introduction

Diabetes mellitus is a chronic metabolic disorder resulting from insulin deficiency, characterized by hyperglycaemia, altered metabolism of carbohydrates, protein and lipids, and an increased risk of vascular complication.[1]

In conventional therapy, type I diabetes is managed with exogenous insulin and type 2 with oral hypoglycemic agents (sulphonylureas, biguanides etc). In traditional practice medicinal plants are used in many countries to control diabetes mellitus. Diabetes mellitus has recently been identified by Indian Council of Medical Research (ICMR) as one of the refractory diseases for which satisfactory treatment is not available in modern allopathic system of medicine and suitable herbal preparations are to be investigated. A large number of plant preparations have been reported to possess antidiabetic activity over last several decades. Researchers in India have documented the use of over 150 plants in various families with hypoglycemic activity.[2]

*Cassia occidentalis* Linn. of family Caesalpiniaceae is a common weed scattered from foothills of Himalayas to West Bengal, South India, Burma, and Sri Lanka. The plant is a diffuse (usually annual) under shrub with loosely spreading branches 60-150 cm long, found throughout India, up to an altitude of 1500 m.[3] Different parts of this plant have been reported to possess anti-inflammatory, antihepatotoxic,[4] antibacterial,[5] and antiplasmodial activities.[6] They possess purgative, tonic, febrifugal, expectorant, and diuretic properties. The plant is also used to cure sore eyes, hematuria, rheumatism, typhoid, asthma, and disorder of hemoglobin and is also reported to cure leprosy. An infusion of the bark is given in diabetes.[3] A wide range of chemical constituents isolated from *C. occidentalis* including sennoside, anthraquinone glycoside,[7] fatty oils, flavonoid glycosides, galactomannan, polysaccharides, and tannins.[8]

In view of alleged antidiabetic potential of *C. occidentalis*, different extracts of the plant on fasting blood sugar levels and biochemical parameters such as serum cholesterol, total protein, and triglyceride were investigated. Histological examination was also carried out on pancreatic tissue of experimental animals.

## Materials and Methods

### Plant Material

The plant of *C. occidentalis* have been collected from Kaaripatti, Salem district, Tamil Nadu, with the help of field botanist. The plant of *C. occidentalis* have been authenticated by Prof. A. Balasubramanion, horticulturist, director of ABS Botanical Conservation, Research and Training Centre, Kaaripatti, Salem district, Tamil Nadu, India (Ref. ABSRTC/08/A-4069). The whole plant was dried initially under shade. It was preserved in a tightly closed container and powdered as per requirements.

### Preparations of Extracts

The dried whole plant was subjected to size reduction to a coarse powder by using dry grinder and passed through sieve. About 150 g of this powder was packed into soxhlet apparatus and extracted successively with petroleum ether, chloroform, and water (yield 1.61%, 1.84%, 1.2%, respectively). The solvent was recovered by distillation in vacuo and extracts were stored in desiccator and used for subsequent experiments.

### Preliminary Phytochemical Screening

Extracts obtained from *C. occidentalis* were subjected to various qualitative tests for the identification of various plant constituents present in this species.[9]

### Animals

Healthy adult male Wistar albino rats between 2 and 3 months of age and weighing about 150-200 g were used for the study. The animals were housed in polypropylene cages, maintained under standard conditions (12 h light: 12 h dark cycle; 25 ± 30°C; 35–60% humidity). They were fed with standard rat pellet diet (Hindustan Lever Ltd., Mumbai, India) and water ad libitum. The Institutional Animal Ethical Committee of VNS, Bhopal (M.P.), India (778/03/c/CPCSEA), approved the study.

### Sample Collection

Blood samples were collected by the retro-orbital plexus puncture method from overnight fasted rats under light ether anesthesia and blood glucose levels were estimated using Accu-chek Active ^TM^ glucose strips in Accu-chek Active ^TM^ Test Meter.

### Acute Toxicity Study

Normal healthy rats were divided into five groups of six animals each. Different doses (100, 250, 500, 750 and 1000 mg/kg body weight) of different extracts (petroleum ether, chloroform and aqueous extract) of plant *C. occidentalis* were administered orally. The rats were observed continuously for 2 h for behavioral, neurological, and autonomic profiles and after 24 and 72 h for any lethality.[10]

### Assessment of Extracts of C. occidentalis on Normal Fasted Rats

For the normoglycemic study, rats were divided into five groups (n=6) and were administered 2% gum acacia solution, metformin(0.5 g/kg),[11] petroleum ether extract, chloroform extract, and aqueous extract (200 mg/kg each), respectively. The blood glucose levels were measured just prior to and 2, 4, and 6 h after drug administration.[12]

### Assessment of Extracts of C. occidentalis on Alloxan-Induced Diabetic Rats

Diabetes was induced in rats by injecting 120 mg/kg of alloxan monohydrate intraperitoneally in 0.9% w/v NaCl to over-night fasted rats. The rats were then kept for the next 24 h on 10% glucose solution bottles, in their cases to prevent hypoglycemia.[13] After 72 h of injection, rats with marked hyperglycemia (fasting blood glucose > 250 mg/dl) were selected and used for the study. The selected diabetic animals were divided into five groups (n = 6). And one more group of normal non-alloxanized animals was also added in the study. Group I (normal control or non-alloxanized rats) and group II (untreated diabetic control rats) received a single oral dose of 0.5 ml/100g of the vehicle; group III diabetic rats were treated orally with metformin (0.5 g/kg) as reference drug. Groups IV, V, and VI were treated orally with petroleum ether, chloroform, and aqueous extract at the dose 200 mg/kg, respectively. Fasting blood glucose estimation was done at 0, 2, 4, and 6 h after treatment. Treatment was continued for 21 consecutive days. The fasting blood glucose levels were estimated on days 0, 1, 7, 14, and 21.[14]

### Estimation of Biochemical Parameters

On day 21, blood was collected from retro-orbital plexus of the overnight fasted rats under light ether anesthesia and kept aside for ½h for clotting. Serum was separated by centrifuging the sample at 6000 rpm for 20 min. The serum was analyzed for total protein (Biuret method),[15] cholesterol (CHOD-PAP method),[16] and triglyceride (GPO method).[17]

### Histopathological Studies

Pancreatic tissues from all groups were subjected to histopathological studies. The whole pancreas from each animal was removed after sacrificing the animal under anesthesia and was collected in 10% formalin solution and immediately processed by the paraffin technique. Sections of 5 *μ*m thickness were cut and stained by hematoxylin and eosin (H and E) for histological examination.[18]

### Statistical analysis

All the values of body weight, fasting blood sugar, and biochemical estimations were expressed as mean ± standard error of mean (S.E.M.). The results are analyzed for statistical significance using one-way ANOVA followed by Dunnett’s test. *P* < 0.05 was considered significant.

## Results

### Preliminary Phytochemical Screening

Preliminary phytochemical screening of the extract of *C. occidentalis* revealed the presence of alkaloids, glycosides, proteins and amino acids, sterols, carbohydrates, phenolic compounds, flavonoids, saponins, and tannins.

### Acute Toxicity Studies

All aqueous-treated rats showed no discernible behavioral changes up to 500 mg/kg by oral route. No mortality was observed at this dose during 72 h observation period.

### Antihyperglycaemic activity screening in normal and alloxan induced diabetic rats

The antidiabetic effects of various extracts of *C. occidentalis* on the fasting blood sugar level of normal and diabetic rats are is shown in Tables 1 and 2. In normal animals, significant (*P*<0.05, *P*<0.01) reduction in the blood glucose level was observed by the aqueous extract as compared to the control [Table 1]. However, treatment of petroleum ether and chloroform extract of *C. occidentalis* could not bring back the sugar to normal levels.

**Table 1:** The effect of various extracts of *C. occidentalis* on blood glucose level in normal rats.

*Group*	*Treatment*	*Blood glucose concentration (mg/dl)*			
**	**	*0 hr*	*2 hr*	*4 hr*	*6 hr*
I	Control (vehicle 2% gum acacia)	87.2 ± 1.1	85.3 ± 0.4	85.1 ± 0.6	86.6 ± 0.3
II	Metformin (0.5 g/kg)	88.5 ± 0.9	61.5 ± 1.2**	56 ± 1.4**	62 ± 2.1**
III	Petroleum ether extract (200mg/kg)	87.5 ± 0.7	86.6 ± 0.6	86 ± 5.8	86.5 ± 0.3
IV	Chloroform extract (200mg/kg)	87.7 ± 2.4	84.4 ± 2.2	83 ± 2.5	84 ± 2.0
V	Aqueous extract (200 mg/kg)	84.5 ± 0.9	79 ± 0.6*	75.8 ± 1.5**	78.4 ± 1.8**

All values are given as mean ± SEM for groups of six animals each

* *P*<0.05;

** *P*<0.01 vs. control (vehicle)

**Table 2:** Effect of various extracts of *C. occidentalis* on blood glucose level in alloxan (120 mg/kg i.p.)-induced diabetic rats.

*Groups*	*Treatment*	*Blood glucose concentration (mg/dl)*							
**	**	*0 hr*	*2 hr*	*4 hr*	*6 hr*	*Day 1*	*Day 7*	*Day 14*	*Day 21*
I	Normal control (vehicle 2 % gum acacia)	87.5 ± 1.6	87.6 ± 1.3	87.9 ± 1.9	88.2 ± 1.5	88.3 ± 1.5	88.8 ± 1.3	89.7 ± 1.9	90.4 ± 1.3
II	Diabetic control (vehicle 2% gum acacia)	262.1 ± 5.3	266 ± 5.1**	269.5 ± 3.7**	270.6 ± 3.1**	272.6 ± 2.7**	280 ± 1.2**	293.6 ± 2.2**	313.2 ± 3.6**
III	Alloxan (120mg/kg, ip) + Metformin (0.5 g/kg)	272.2 ± 6.4	239.6 ± 1.9*	228.4 ± 2.3**	221.3 ± 1.5**	208.1 ± 3.6**	165.3 ± 4.2**	107.7 ± 3.2**	95.5 ± 1.4**
IV	Alloxan (120mg/kg, ip) + Petroleum ether extract (200mg/kg)	272.3 ± 5.8	271.7 ± 5.5	270.7 ± 5.4	270.3 ± 5.8	269.7 ± 5.5	270.1 ± 6.1	270 ± 5.2*	269.3 ± 4.9**
V	Alloxan (120mg/kg, ip) + Chloroform extract (200mg/kg)	278.7 ± 7.9	273 ± 7.0	269.4 ± 7.3	266.2 ± 7.6	256.8 ± 6.4	242.4 ± 4.8**	230.7 ± 5.2**	221.4 ± 4.3**
VI	Alloxan (120mg/kg, ip) + Aqueous extract (200mg/kg)	245.3 ± 11.8	235.1 ± 10.4**	229.3 ± 10.1**	224.3 ± 9.5**	214.3 ± 11.2**	190.4 ± 9.7**	170.7 ± 5.8**	151.6 ± 2.6**

All values are expressed as mean ± SEM (n=6); Group II was compared with Group I; Groups III-VI were compared with group II;

* *P*<0.05;

** *P*<0.01

Acute and chronic treatment of the aqueous extract of *C. occidentalis* (200 mg/kg) in alloxan-induced diabetic rats resulted in a significant (*P*<0.01) decrease in the elevated blood glucose levels as compared to the control. Acute treatment of petroleum ether and chloroform extract of *C. occidentalis* could not bring back the sugar to normal levels. However in repeated dose treatment, petroleum ether extract showed significant anti-hyperglycemic activity from day 14 and chloroform extract showed significant anti-hyperglycemic activity from day 7.

### Biochemical Parameters

Significant differences were observed in serum lipid profiles (cholesterol and triglyceride) and serum protein [Table 3] in aqueous extract (200 mg/kg)-treated diabetic animals, when compared with the diabetic control and normal animals (*P* < 0.01).

**Table 3:** Effects of Aqueous extract of *C. occidentalis* on some biochemical parameters in alloxan-induced diabetic rats.

*Groups*	*Treatment*	*Cholesterol (mg/dl)*	*Triglyceride (mg/dl)*	*Total Protein (g/dl)*
I	Normal control (vehicle)	86.65 ± 1.83	85.62 ± 3.22	7.20 ± 0.23
II	Diabetic control (vehicle)	153.88 ± 4.19**	189.86 ± 1.74**	4.73 ± 0.53**
III	Alloxan (120mg/kg, ip) + Metformin (0.5 g/kg)	92.03 ± 1.09**	94.13 ± 1.20**	6.95 ± 0.07**
IV	Alloxan (120mg/kg, ip) + Aqueous extract (200mg/kg)	135.32 ± 4.04**	145.18 ± 3.67**	6.05 ± 0.08**

All values are expressed as mean ± SEM (n=6); Group II is compared with Group I. Groups III and IV are compared with group II;

** *P*<0.01

### Histopathological studies

**Figure 1:**
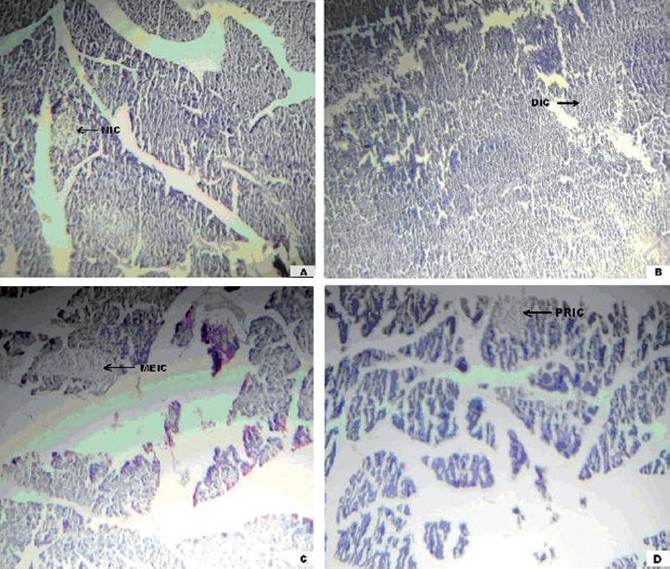
(A) Photomicrographs of normal healthy control group rat showing normal globules of acini with normal islet cells (NIC), stained with H & E. (B) Photomicrographs of diabetic control group rat showing damaged islet cells (DIC) stained with H & E. (C) Photomicrographs of standard (metformin 0.5 g/mg) treated group rat showing moderate expansion of islet cells (MEIC), stained with H & E. (D) Photomicrographs of Aq extract (200 mg/kg)-treated group rat showing partial restoration of islet cells (PRIC) stained with H & E (400X).

Figure 1(A-D) depicts the islets of the pancreas of rats in different groups. Photomicrographs (A) of the normal healthy control group showed normal acini and normal cellular population of the islets of Langerhans. However, in the alloxan only treated rats, there was extensive damage of the islets of Langerhans and they appeared to be irregular (B). Treatment of diabetic rats with metformin showed moderate expansion of cellular population and size of islet cells (C). However, aqueous extract (200 mg/kg) treated-diabetic rats showed partial restoration of normal cellular population and size of islet cells (D).

## Discussion

In light of the results, our study indicates that aqueous extract of *C. occidentalis* exhibited significant anti-hyperglycemic activity in normal and alloxan-induced hyperglycemic rats. In normal rats, administration of aqueous extract showed 6.50%, 10.29%, and 7.21% decline in the blood glucose levels on 2, 4, and 6 h, respectively. Alloxan-induced diabetic rats administered with aqueous extract showed 4.15%, 6.52%, and 8.56% decline in the blood glucose level on 2, 4, and 6 h, respectively, whereas they showed 12.63%, 22.38%, 30.41%, and 38.19% decline in the blood glucose level on 1, 7, 14, and 21 day, respectively. They can also improve the condition of diabetes as indicated by parameters like serum cholesterol, serum triglyceride, and total protein.

It is now established that there is a gradual decrease in beta-cell function and mass that may occur in individuals at high risk of developing type II diabetes. To prevent the loss of beta-cell function and mass, beta-cell stabilization or regeneration must occur.[19] The renewal of β-cells in diabetes has been studied in several animal models. For example epicatechin has been shown to act by β-cell regeneration.[20] Similarly *Vinca rosea* extracts also cause regeneration of β-cell in alloxan-induced diabetic rats.[21]

Progression of type II diabetes is mainly due to loss of pancreatic β-cell function, which results in increased impairment of patient’s ability to produce insulin in response to increased blood glucose. Metformin directly improves insulin action and is effective only in the presence of insulin.[22] It is to be seen whether the antidiabetic effect of *C. occidentalis* may be due to increased insulin secretion, similar to that observed in metformin.

In our studies, damage of pancreas was observed in alloxan-treated diabetic control rats [Figure 1B]. The metformin-treated group showed regeneration of β-cells [Figure 1C]. The comparable regeneration was also shown by aqueous extracts of *C. occidentalis* [Figure 1D]. Photomicrographs reinforce healing of pancreas by the aqueous extract of *C. occidentalis*, as a plausible mechanism of their antidiabetic activity. The antidiabetic activity of *C. occidentalis* may be due to the presence of flavonoids. It is reported that flavanoids constitute the active biological principles of most medicinal plants with hypoglycemic and antidiabetic properties.[23] However the extract should further be subjected to bioactivity-guided drug discovery to isolate the lead compound responsible for antidiabetic activity and possible mechanisms(s) of action.

In conclusion, *C. occidentalis* exhibited significant antihyperglycemic activities in normal and alloxan-induced diabetic rats. The aqueous extract of *C. occidentalis* also showed improvement in lipid profile as well as regeneration of β-cell of pancreas and so might be of value in treatment of diabetes.
